# Supplementary material for: HLA-DR Expression on Monocytes and Sepsis Index Are Useful in Predicting Sepsis
Source: Biomedicines. 2023 Jun 26;11(7):1836. doi: 10.3390/biomedicines11071836 (PMC10377223; doi:10.3390/biomedicines11071836)
Supplement: Supplementary file 1 [file biomedicines-11-01836-s001.zip › biomedicines-2422214-supplementary.pdf]

Supplementary Table 1. Candidate biomarkers for predictive outcome in total cohort of patients.

Data measured	Total cohort n=77					
	Basal	Day +3	Day+6	Day +9	Day +12	Day +15
PCR (mg/ml)	71.90±76.71	161.83±133.42	153.76±151.02	109.99±71.82	125.78±136.53	144.05±172
Lymphocyte count (cell/uL)	1261±646	1190±640	1302±627	1449±1243	1649±1351	1737±1459
Percentage of CD14+ HLA-DR+ monocytes (%)	83.94±16.52	83.72±15.35	83.59±16.8	85.98±15.54	87.08±13.44	85.26±18.99
MFI of CD14+ HLA-DR+ monocytes	1941±1206	2218±1710	2253±1398	2792±2121	2791±2421	2779±1699
HLA-DR Index	1.55±0.91	1.81±1.45	1.16±1.02	1.26±1.15	1.42±0.82	1.47±0.77
Sepsis Index	0.11±0.13	0.16±0.18	0.12±0.15	0.1±0.12	0.15±0.17	0.65±0.89

Supplementary Table 2. Candidate biomarkers for predictive outcome in Septic and Non-Septic patients.

	Septic cohort					
	**Basal n=55**	**Day +3 n=55**	**Day+6 n=48**	**Day +9 n=45**	**Day +12 n=39**	**Day +15 n=24**
**PCR (mg/ml)**	80.16±78.07	182.9±132.9	171.9±155	124.8±68.39	132.88±138.23	155.7±189.63
**Lymphocyte count (cell/uL)**	1235±645	1091±529	1312±633	1515±1320	1711±1386	800.36±523.80
**Percentage of CD14+ HLA-DR+ monocytes (%)**	82.42±17.9	81.7±16.22	81.29±18.01	86.35±11.48	87.01±13.74	80.54±21.56
**MFI of CD14+ HLA-DR+ monocytes**	1902±1223	1933±1198	2026±1241	2634±1953	2603±1786	1873±1396
**HLA-DR Index**	1.50±0.97	1.76±1.59	1.23±1.14	1.32±1.19	1.45±0.80	1.28±0.91
**Sepsis Index**	0.13±0.14	0.19±0.19	0.15±0.17	0.11±0.13	0.15±0.17	1.03±0.48
	**Non-Septic cohort**					
	**Basal n=22**	**Day +3 n=22**	**Day+6 n=22**	**Day +9 n=13**	**Day +12 n=9**	**Day +15 n=1**
**PCR (mg/ml)**	44.84±68.93	93.46±117.6	38.73±12.13	30.77±12.76	137.64±105.85	85,8
**Lymphocyte count (cell/uL)**	1266±625	1474±862	1251±657	915±534	857±448	500
**Percentage of CD14+ HLA-DR+ monocytes (%)**	87.22±12.36	88.53±12.13	89.08±11.93	93.67±6.3	85.1±12.47	83
**MFI of CD14+ HLA-DR+ monocytes**	2046±1214	2942±2552	2689±1541	2865±2022	2079±1976	1570
**HLA-DR Index**	1.69±0.75	1.95±1.04	0.95±0.58	0.93±0.97	1.26±0.96	1,34
**Sepsis Index**	0.06±0.05	0.08±0.08	0.06±0.05	0.04±0.02	0.18±0.16	0,74
	**p-value**					
	**Basal**	**Day +3**	**Day+6**	**Day +9**	**Day +12**	**Day +15**
**PCR (mg/ml)**	0.140	**0.030**	0.050	**0.030**		
**Lymphocyte count (cell/uL)**	0.870	0.140	0.690	0.090	0.050	
**Percentage of CD14+ HLA-DR+ monocytes (%)**	0.670	**0.040**	0.070	0.060	0.660	
**MFI of CD14+ HLA-DR+ monocytes**	0.560	0.110	0.160	0.760	0.200	

**HLA-DR Index**	0.110	0.170	0.550	0.130	0.600	
**Sepsis Index**	0.090	**0.010**	**0.020**	**0.030**	0.610	
